# Loss of DEK Expression Induces Alzheimer’s Disease Phenotypes in Differentiated SH-SY5Y Cells

**DOI:** 10.3389/fnmol.2020.594319

**Published:** 2020-11-16

**Authors:** Allie N. Greene, Lois G. Parks, Matia B. Solomon, Lisa M. Privette Vinnedge

**Affiliations:** ^1^Neuroscience Graduate Program, University of Cincinnati College of Medicine, Cincinnati, OH, United States; ^2^Division of Oncology, Cancer and Blood Diseases Institute, Cincinnati Children’s Hospital Medical Center, Cincinnati, OH, United States; ^3^Department of Psychology, University of Cincinnati, Cincinnati, OH, United States; ^4^Department of Pediatrics, University of Cincinnati College of Medicine, Cincinnati, OH, United States

**Keywords:** tau, neurite formation, DEK, dementia—Alzheimer disease, tau phosphorylation

## Abstract

Alzheimer’s disease (AD) is the most common cause of dementia and is characterized by the buildup of β-amyloid plaques and neurofibrillary Tau tangles. This leads to decreased synaptic efficacy, cell death, and, consequently, brain atrophy in patients. Behaviorally, this manifests as memory loss and confusion. Using a gene ontology analysis, we recently identified AD and other age-related dementias as candidate diseases associated with the loss of DEK expression. DEK is a nuclear phosphoprotein with roles in DNA repair, cellular proliferation, and inhibiting apoptosis. Work from our laboratory determined that DEK is highly expressed in the brain, particularly in regions relevant to learning and memory, including the hippocampus. Moreover, we have also determined that DEK is highly expressed in neurons. Consistent with our gene ontology analysis, we recently reported that cortical DEK protein levels are inversely proportional to dementia severity scores in elderly female patients. However, the functional role of DEK in neurons is unknown. Thus, we knocked down DEK in an *in vitro* neuronal model, differentiated SH-SY5Y cells, hypothesizing that DEK loss would result in cellular and molecular phenotypes consistent with AD. We found that DEK loss resulted in increased neuronal death by apoptosis (i.e., cleaved caspases 3 and 8), decreased β-catenin levels, disrupted neurite development, higher levels of total and phosphorylated Tau at Ser262, and protein aggregates. We have demonstrated that DEK loss *in vitro* recapitulates cellular and molecular phenotypes of AD pathology.

## Introduction

Dementia affects 5–8% of the population worldwide over 60 years old (World Health Organization, 2017); this number is as high as 14% in the USA (Alzheimer’s Association, 2019a). Dementia is characterized by memory loss, personality changes, and impaired cognitive function. Alzheimer’s disease (AD) is the most common cause of dementia, accounting for 60–80% of cases (Alzheimer’s Association, 2019a). Early-onset, genetic AD is modeled widely in research but is less common in the human population. However, late-onset, sporadic AD accounts for around 99% of patients (Alzheimer’s Association, 2019b). There is no cure for AD, and very few effective treatments, due in part to an incomplete understanding of the cause(s) of late-onset AD. Many biological and environmental factors are known to contribute to the risk of AD and associated dementias, including biological sex (Seshadri et al., 1997; Mazure and Swendsen, 2016), race (Chen and Zissimopoulos, 2018), vascular conditions (O’Brien and Markus, 2014), metabolic conditions (Craft, 2009), an education level (Karp et al., 2004), a history of depression (Ownby et al., 2006) and more. While the causes of AD are still unclear, the physiological consequences of the disease are more explicit. Ultimately, AD results in decreased synaptic communication between neurons due to the buildup of β-amyloid plaques between cells and neurofibrillary tangles containing Tau within cells. This leads to cell death and eventually brain atrophy in patients.

The neurodegeneration caused by AD results in cellular and molecular anomalies, such as DNA damage, cell death, plaques, and tangles. DNA damage and cell death are observed in dementia, while β-amyloid plaques and Tau tangles are unique to AD. Tau stabilizes microtubules and is essential to maintain their normal functions, such as axonal transport, neurite outgrowth, and maintaining neuronal morphology (Johnson and Stoothoff, 2004). When Tau is hyperphosphorylated, it dissociates from microtubules and can lead to microtubule dysfunction and destabilization, the formation of Tau filaments, and cell death (Johnson and Stoothoff, 2004; Ramkumar et al., 2018). Phosphorylation of Tau at different sites can alter its function. For example, phosphorylation at serine 262, within the microtubule-binding domain of Tau, most strongly attenuates Tau binding to microtubules (Fischer et al., 2009; Haj-Yahya et al., 2020).

Our group was the first to associate central nervous system-relevant diseases, such as AD, with decreased expression of the chromatin-binding protein DEK (Ghisays et al., 2018). Still, the cellular and molecular relevance of DEK expression in the brain is largely unexplored. Previous work using peripheral tissues have identified DEK as a nuclear, chromatin-associated phosphoprotein that promotes cellular proliferation, aids in DNA damage repair, and prevents apoptosis (Waldmann et al., 2002; Wise-Draper et al., 2006; Khodadoust et al., 2009; Kavanaugh et al., 2011; Privette Vinnedge et al., 2011, 2015; Broxmeyer et al., 2012; Koleva et al., 2012; Waidmann et al., 2014; Smith et al., 2017). The majority of previous reports have focused on the role of DEK in solid tumors and hematologic malignancies, autoimmune diseases, and hematopoiesis. DEK is overexpressed in a majority of solid tumors (Sanchez-Carbayo et al., 2003; Grasemann et al., 2005; Wu et al., 2008; Khodadoust et al., 2009; Liu et al., 2012; Privette Vinnedge et al., 2015), and is also known to be an autoantigen in autoimmune disorders such as juvenile idiopathic arthritis (Sierakowska et al., 1993; Dong et al., 2000; Mor-Vaknin et al., 2011). High levels of DEK expression in human and murine cells can have oncogenic consequences, but substantially overexpressed DEK in normal cells could lead to cell death, such as a rough eye phenotype in *Drosophila* (Lee et al., 2008; Pease et al., 2015). DEK loss, or low expression of DEK, has not been widely studied, but some reports suggest that DEK loss leads to a reduced inflammatory response (Kim et al., 2015; Mor-Vaknin et al., 2017), increased DNA damage (Smith et al., 2017), and apoptosis (Smith et al., 2017). Most recently, Miao et al. (2020) found that microRNA-138, known to be associated with AD, decreases DEK expression in SH-SY5Y cells, the same cell culture model used here.

In 2018, a gene ontology analysis by our group found that AD, dementia, and other age-associated anomalies had transcriptomic signatures associated with DEK loss in human cells (Ghisays et al., 2018). Also, using human postmortem brain samples we found that lower DEK protein expression in the anterior cingulate cortex was correlated with increasing dementia severity in women, but not men (O’Donovan et al., 2018). These data lead us to postulate that nuclear DEK could be neuroprotective. DEK is expressed throughout the brain, including in regions important for learning and memory, and DEK is likely expressed in multiple cell types, including neurons, microglia, and astrocytes (Ghisays et al., 2018). However, the functional role of DEK in neurons remains unknown. Here, we use an *in vitro* model, the SH-SY5Y cell line that can be differentiated to assume a neuronal morphology and phenotype. SH-SY5Y cells are widely used in the AD field (Boyle et al., 2012; Koriyama et al., 2015; Oguchi et al., 2017; Pascual-Caro et al., 2018; Shang et al., 2019). Their ability to be differentiated makes them appealing because they phenotypically and molecularly mimic primary neurons after going through the differentiation process (Gimenez-Cassina et al., 2006; Cheung et al., 2009; Agholme et al., 2010; Xie et al., 2010; Shipley et al., 2016). This allows us to study, for the first time, the cellular and molecular consequences of DEK loss in neuron-like human cells. We hypothesized that DEK loss *in vitro* would result in cellular and molecular signatures of dementia and AD. Indeed, we found that DEK loss in SH-SY5Y cells results in apoptosis, aberrant neurite formation, and increased total Tau expression. Further, we see increased expression of phosphorylated Tau with DEK loss, specifically within the microtubule-binding domain of the Tau protein at Ser262. This suggests that DEK may be important for the normal function of microtubules and that DEK loss recapitulates phenotypes observed in AD, such as hyperphosphorylated Tau and neuronal death by apoptosis.

## Materials and Methods

### Cell Culture and Viral Transduction

SH-SY5Y cells were purchased from ATCC and cultured in 50% Minimum Essential Medium (MEM) and 50% F-12 with 10% fetal bovine serum. Cell counts were performed using Trypan blue exclusion and a Countess II automated cell counter (Life Technologies). For viral transduction, HEK293T cells were transfected with either non-targeting shRNA (NTsh; control) or DEK-targeting shRNA (DEKsh; pLKO.1_DEK832; #TRCN0000013104) plasmid DNA in the pLKO.1 plasmid backbone (Sigma–Aldrich MISSION shRNA), as previously published (Privette Vinnedge et al., 2011). The virus was collected from HEK293T cells 48–72 h later, filtered with a 0.45-micron syringe filter, and added to SH-SY5Y cells overnight with polybrene. The selection for transduced cells was completed using puromycin (2.0 μg/ml) for 72 h.

### Neuronal Differentiation

SH-SY5Y cells were differentiated according to the protocol by Shipley et al. (2016). Briefly, cells were plated at 50,000 cells/ml to uncoated 10 cm culture plates and incubated in media with heat-inactivated fetal bovine serum (FBS; Seradigm). As the differentiation progressed, media which contained retinoic acid (RA; Sigma–Aldrich; 10 μM final concentration) and less FBS (15% reduced to 0%) was used. On day 10 of differentiation, the cells were transferred to 10 cm plates coated with extracellular matrix (ECM; Sigma–Aldrich) for Western blot and PCR and to coverslips coated with poly-L-lysine (Sigma–Aldrich) for immunofluorescence. The final media changes exposed the cells to neuronal growth factors such as BDNF (Sigma–Aldrich), as well as B-27 supplement (Thermo Fisher Scientific, Waltham, MA, USA), potassium chloride, and dibutyryl cyclic AMP (Santa Cruz Biotechnologies). Differentiated neuronal cells were harvested on day 18 for immunofluorescence, Western blot, and qPCR analyses. Phase-contrast images were collected using a Leica DMIL microscope and SPOT imaging software. Neurite number was determined manually. In ImageJ, neurites were traced to measure length, and cell bodies were outlined to measure area. Sholl analysis was conducted using the Simple Neurite Tracer plugin from FIJI/ImageJ.

### Immunocytochemistry/Immunofluorescence

Differentiated cells on coated poly-L-lysine coverslips were fixed with 4% paraformaldehyde. After washing with PBS, cells were permeabilized with 0.1% Triton X-100 for 5 min, then washed with PBS again. Cells were blocked in 5% normal goat serum for 1 h at room temperature and then incubated in primary antibody overnight at 4°C. Primary antibodies included DEK (1:100, BD Biosciences, mouse), cleaved caspase 3 (CC3; 1:400, Cell Signaling, rabbit), alpha-Tubulin (1:500; Cell Signaling, mouse), Tau-5 (total Tau, 1:100; Abcam, mouse), Tau-1 (unphosphorylated Tau, 1:200, Millipore, mouse) Phosphorylated Tau S262 (1:100; Abcam, rabbit), AT8 (S202/S205; 1:100; Invitrogen, mouse monoclonal), AT180 (T231/S235; 1:50; Invitrogen, mouse monoclonal), and clone PHF-1 (S396/S404; 1:50; Invitrogen, rabbit). Coverslips were washed in PBS and incubated in secondary antibodies (1:250; Alexa Fluor 488 or 568, mouse or rabbit; Abcam) for 1 h at room temperature. The Proteostat Aggresome Detection Kit (Enzo) was used in place of a primary antibody on a subset of samples to determine if protein aggregates were present in the cells. Cells were washed once more before the coverslips were mounted onto microscope slides using Prolong Gold antifade reagent with DAPI (Invitrogen). Slides were stored in the dark at room temperature. Images were taken on a Nikon A1 inverted confocal microscope at 20× for fluorescence intensity quantification, and at 60× for qualitative images. Two to three biological replicates and 14–20 2D fields of view (single confocal images; 45–100 cells per image) were analyzed for fluorescent intensity using optical density in ImageJ.

### Western Blot

Undifferentiated cells were collected *via* trypsinization and centrifugation. Differentiated neuron-like cells, which are loosely adherent, were collected by rinsing plates with sterile PBS and subsequent centrifugation. Proteins were separated by SDS–PAGE and transferred to a PVDF membrane. The membrane was blocked in 5% milk solution in TNET and incubated in primary antibodies including β-catenin (D10A8, 1:1,000, Cell Signaling), active β-catenin (non-phospho S45, 1:1,000, Cell Signaling) CC3 D175 (1:1,000; Cell Signaling), caspase 8 (1C12, 1:1,000; Cell Signaling), phosphorylated Ser 15 p53 (1:1,000; Cell Signaling), AT180 (1:500; Invitrogen) AT8 (1:500; Invitrogen), Phosphorylated Tau S262 (1:1,000; Abcam), PHF-1 (1:1,000; Invitrogen), DEK (1:1,000; ProteinTech rabbit polyclonal or 1:1,000 BD Biosciences, mouse monoclonal), Tau-1 (1:1,000; Sigma–Aldrich, mouse), Tau-5 (1:1,000; Abcam, mouse), and Actin C4 (1:10,000; mouse; gift of James Lessard, Cincinnati Children’s Hospital, available at Seven Hills Bioreagents). Secondary HRP-conjugated antibodies for mouse (1:2,500) or rabbit (1:3,000) were used (Cytiva Lifescience), and blots were imaged using ECL reagents and the BioRad ChemiDoc Touch imaging system. Densitometry was determined using Image Lab. The band intensity of each sample was normalized to the actin loading control. Fold changes in phosphorylated Tau relative to total Tau (Tau-5) was calculated by normalizing phosphorylated Tau densitometry values to values of total Tau within the same group (three to four biological replicates per group).

### qRT-PCR

Cell pellets were collected as described above. RNA was extracted using the Qiagen RNeasy Mini kit and cDNA was synthesized using the QuantiTect Reverse Transcription kit (Qiagen). Real-time PCR analysis to determine expression levels of *Tau* (F, 5′-GATTGGGTCCCTGGACAATA-3′; R, 5′-GTGGTCTGTCTTGGCTTTGG-3′) and *Dek* (F, 5′-TGTTAAGAAAGCAGATAGCAGGACC-3′; R, 5′-ATTAAAGGTTCATCATCTGAACTATCCTC-3′) was performed using SYBR Green PCR master mix (Invitrogen) in the StepOnePlus Real-Time PCR System (Applied Biosystems). Importantly, *Tau* primers were designed to recognize all isoforms of *Tau* mRNA. All measurements were done in triplicate and the target gene expression levels were normalized to β-actin expression. The increase or decrease in target genes expression was determined using the ΔΔC_T_-method.

### Statistics

Comparisons between NTsh and DEKsh samples were graphed with GraphPad Prism (version 8) and analyzed for statistical significance using Student’s *t*-test, two-tailed.

## Results

### DEK Knockdown in SH-SY5Y Cells Causes Apoptotic Cell Death

**Figure 1:**
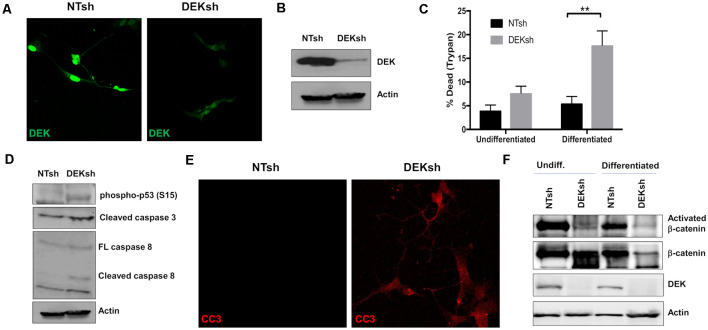
DEK knockdown in SH-SY5Y cells increases cell death *via* apoptosis. **(A,B)** DEK is successfully knocked down in SH-SY5Y cells using an shRNA lentiviral construct. A non-targeted shRNA (NTsh) was used as a control. **(C)** Number of dead cells were counted using Trypan blue. Neuronal differentiation exacerbated cell death due to DEK loss (**p < 0.01). **(D)** DEKsh cells showed increased phosphorylated p53 and cleaved caspase 3 (CC3) and 8, indicative of apoptosis. **(E)** Representative immunofluorescence images display increased CC3 in DEKsh cells. **(F)** Total β-catenin expression levels decreased in differentiated DEKsh cells. Activated β-catenin is downregulated in both undifferentiated and differentiated DEKsh cells.

To study the loss of DEK *in vitro*, we transduced SH-SY5Y cells with lentivirus containing shRNA to target DEK and non-targeting (NT) shRNA as a control. DEK was successfully knocked down in these cells, as confirmed with immunofluorescence (IF; Figure 1A) and Western blot (Figure 1B). DEK loss is known to cause cell death, and indeed we observed increased cell death in DEKsh cells as determined by Trypan blue exclusion. This was further exaggerated after neuronal differentiation (Figure 1C; *p* < 0.01). Notably, we did not observe differences in the cell cycle profiles between NTsh and DEKsh cells (data not shown). DEK is important for DNA repair and preventing apoptosis in cancer cells. Consistent with this, we see increasing evidence of cellular stress, as determined by increased phosphorylated p53, and increased expression of the apoptotic markers CC3 and cleaved caspase 8 in DEKsh cells compared to NTsh controls (Figures 1D,E). Previously, we have shown that DEK expression promotes Wnt/β-catenin signaling. Consistent with these previous findings, we noted decreased levels of activated β-catenin with DEK knockdown in both undifferentiated and differentiated cells. Total β-catenin was decreased in DEKsh cells only after differentiation (Figure 1F).

### DEK Knockdown Impairs Neurite Formation in Differentiated Cells and Results in Tau Hyperphosphorylation and Accumulation

**Figure 2:**
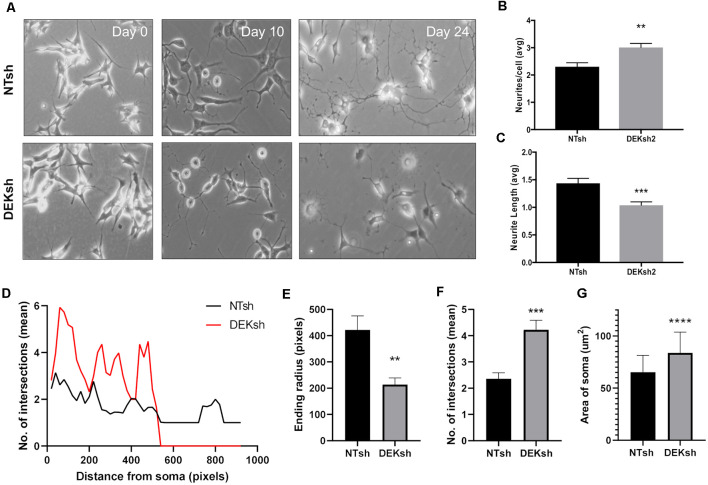
DEK loss impairs neurite formation in differentiated cells. **(A)** Representative images from the progression of SH-SY5Y cells into differentiated neurons. **(B)** Quantification of the number of neurites per cell in **(A)**. DEKsh had significantly more neurites per cell (**p < 0.01), but **(C)** the neurites from DEKsh cells were shorter in length (***p < 0.001). **(D)** Sholl analysis results represent the number of intersections between neurites and radii projecting from the focal point (center of cell body) generated by Simple Neurite Tracer Sholl analysis in ImageJ. **(E)** According to Sholl analysis, neurites on DEKsh cells were significantly shorter than NTsh (**p < 0.01). **(F)** Quantification of the mean number of intersections shows increased intersections for DEKsh cells compared to controls, as determined by Sholl analysis (***p < 0.001). **(G)** The cell body (soma) sizes of DEKsh cells were significantly greater than NTsh (****p < 0.0001).

We found that the growth of neurites, which can give rise to either axons or dendrites in neurons, was impaired by DEK loss throughout the differentiation process (Figure 2A). The number of neurites per cell and the length of the neurites were quantified. DEKsh cells had more neurites per cell, but the neurites were shorter (Figure 2B, *p* < 0.01; Figure 2C, *p* < 0.001). This was corroborated by Sholl analysis of differentiated SH-SY5Y cells, in which the ending radius of DEKsh cells was significantly lower than NTsh (Figures 2D,E, *p* < 0.01), while the number of intersections of neurites with radii generated by Sholl analysis was greater in DEKsh cells (Figures 2D,F, *p* < 0.001). An additional morphology difference was noted in a larger soma size in DEKsh cells (Figure 2G, *p* < 0.0001). Importantly, a dysfunction in neurite outgrowth could be due to decreased microtubule stability (Dent and Gertler, 2003; Athamneh et al., 2017). We then decided to focus on the protein Tau, which is known to stabilize microtubules and is abnormally phosphorylated in AD, leading to the formation of Tau aggregates, or “tangles.”

**Figure 3:**
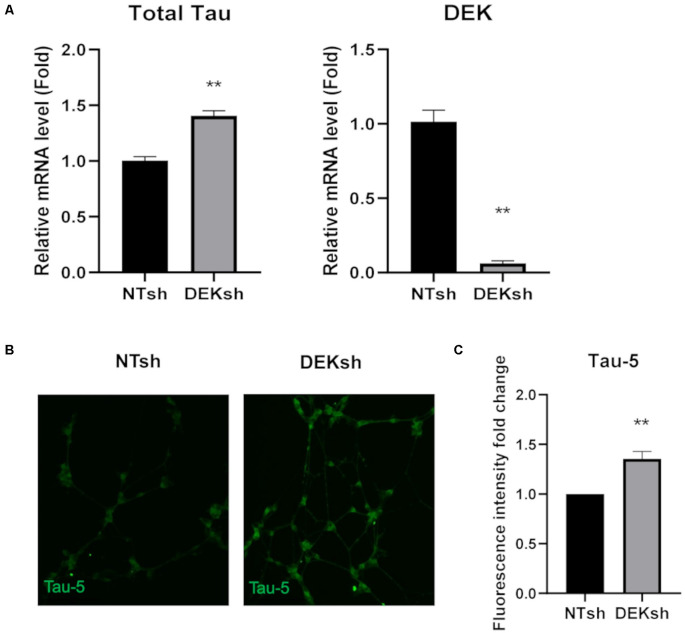
DEK loss causes increased unphosphorylated Tau expression in differentiated SH-SY5Y cells. **(A)** Relative mRNA expression of *Tau* is increased in DEKsh cells, while DEK is confirmed to be transcriptionally downregulated (**p < 0.01). **(B,C)** The fluorescent intensity of the total Tau (Tau-5) expression is significantly higher in DEKsh cells (**p < 0.01).

**Figure 4:**
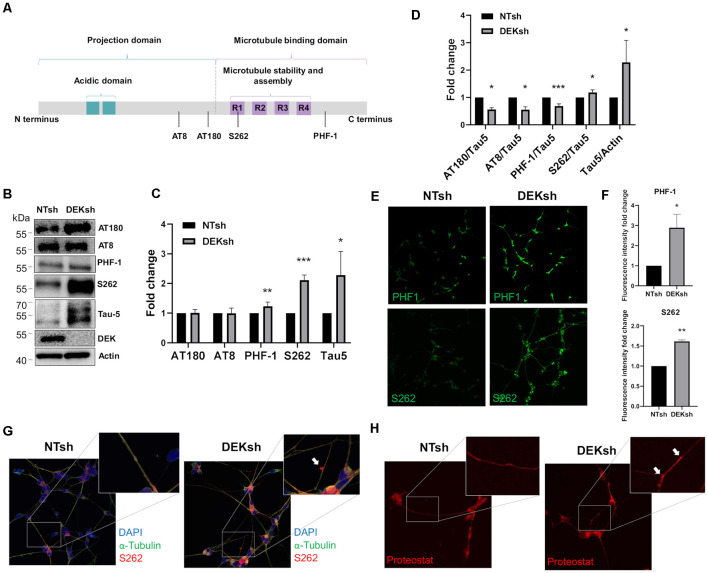
DEK loss increases phosphorylation of Tau at sites within the microtubule-binding domain. **(A)** Representative figure of the longest isoform of Tau (2N4R). The four phosphorylation sites of interest are indicated by a black line. **(B,C)** Western blotting reveals a significant increase in phosphorylated Tau at PHF-1 and S262 in DEKsh cells (*p < 0.05; **p < 0.01; ***p < 0.001). **(D)** When normalized to total Tau (Tau-5) expression levels by western blot **(B)**, S262 was abnormally phosphorylated after DEK knockdown, while Tau at sites AT180, AT8, and PHF-1 were not hyperphosphorylated relative to total Tau (*p < 0.05; **p < 0.01; ***p < 0.001). **(E,F)** Phosphorylation at sites PHF-1 and S262 is increased in DEKsh cells relative to NTsh cells, quantified by fluorescent intensity (*p < 0.05; **p < 0.01). **(G)** Aggregates of S262-phosphorylated Tau are found in the neurites of DEKsh cells, but not in NTsh cells. **(H)** Protein aggregates are observed in DEKsh cells, but not in NTsh cells, using Proteostat staining.

DEK knockdown in differentiated cells increased *Tau* mRNA expression, as observed *via* qRT-PCR using a primer set that identifies all *Tau* isoforms (Figure 3A; *p* < 0.01). Furthermore, immunofluorescence indicated an upregulation of total Tau (Tau-5) in DEKsh cells compared to controls (Figures 3B,C). Western blotting and immunofluorescence revealed increased amounts of unphosphorylated Tau at the epitope recognized by the Tau1 antibody (S195, 198, 199, 202) in DEKsh cells (Supplementary Figure 1A–C; *p* < 0.01). Increased levels of total Tau are found in AD (Sjögren et al., 2001), but an even more definitive marker of pathology in AD is the hyperphosphorylation of Tau (Sjögren et al., 2001; Gong and Iqbal, 2008; Miao et al., 2019). We analyzed total (Tau-5) and phosphorylated Tau levels at multiple phosphorylation sites (Figure 4A). We observed an approximately 2-fold increase in total Tau protein by western blotting, further confirming the upregulation observed by qRT-PCR and immunofluorescence (Figures 4B,C). Next, we tested the levels of potentially pathogenic levels of phosphorylated Tau. Sites within the projection domain of Tau, AT180 (T231/S235) and AT8 (S202/T205), were not abnormally phosphorylated after DEK loss (Figure 4C and Supplementary Figure 1D,E). Interestingly, in DEKsh cells, we observed increased Tau phosphorylation at Serine 262 and PHF-1 (S396/S404) by both western blotting and immunofluorescence, which are all located within the microtubule-binding domain of Tau (Figures 4C,E–G; *p < 0.05; **p < 0.01; ***p < 0.001). We then normalized the levels of phosphorylated Tau to total Tau. In this comparison, the ratio of pTau/Tau for sites in the projection domain (AT8, AT180) and PHF-1 was down-regulated in DEKsh cells, while S262 was modestly, but significantly, upregulated (Figures 4D–F; *p < 0.05; **p < 0.01; ***p < 0.001). Combined, this indicates that the majority of upregulated Tau protein in DEKsh cells is hyperphosphorylated at site S262. This suggests that DEK may be important for maintaining the microtubule-stabilizing properties of Tau. Also, only in DEKsh cells did we find potential aggregates of phosphorylated Tau at Ser262 on neurites (Figure 4G). This implicates DEK’s role in maintaining a balance of physiologically normally phosphorylated Tau; when Tau is hyperphosphorylated, it can aggregate into filaments and tangles within neurons (Lee et al., 1991; Morishima-Kawashima et al., 1995; Lim et al., 2014; Šimić et al., 2016; Alonso et al., 2018). To confirm this staining pattern was not an artifact, we stained differentiated SH-SY5Y cells with Proteostat, which labels protein aggregates. Indeed, we observed similar punctate staining on neurites only in DEKsh cells (Figure 4H), suggesting that there are protein aggregates in DEKsh neuronal cells that were not observed in control cells.

## Discussion

AD is a debilitating global public health crisis on the rise, with still no cure and no infallible preventative measures or treatments. This highlights the importance of identifying novel potential mechanisms by which AD manifests in the brain to further our understanding of the disease and identify drug targets. Before the current study, we found that AD and dementia are candidate DEK loss-associated diseases (Ghisays et al., 2018) and that DEK expression decreased with dementia severity in elderly women (O’Donovan et al., 2018). Here, we are the first to elucidate a potential mechanism for how DEK loss may increase the risk of AD.

As expected, based on previous reports of DEK, loss of the chromatin-binding protein increased the incidence of apoptotic cell death. Additionally, β-catenin, part of the Wnt signaling pathway important for cellular proliferation (Bilir et al., 2013; Privette Vinnedge et al., 2015; Mu et al., 2019), was downregulated after DEK loss. The loss of canonical Wnt signaling and β-catenin induces neuronal apoptosis (Inestrosa and Toledo, 2008). Further, Wnt signaling has been studied as a potential therapeutic target in AD; loss of Wnt signaling exacerbates Tau hyperphosphorylation and β-amyloid deposition and aggregation (Tapia-Rojas and Inestrosa, 2018).

Neuronal differentiation of the control NTsh SH-SY5Y cells resulted in complete neurite networks in which cells were able to make connections with others nearby. The DEKsh cells, however, did not fare as well during the differentiation process. Overall, cells in which DEK was knocked down grew more neurites per cell, but these neurites did not always successfully make connections with other cells; the neurites of DEKsh cells were shorter than those of the controls. This could be due to deficiencies in Tau protein expression or function. Tau is a microtubule-stabilizing protein, critical for the normal function of microtubules (Johnson and Stoothoff, 2004). We found that total Tau mRNA and protein were upregulated in differentiated DEKsh cells. To determine if DEK loss influenced the pathological state of Tau, we examined the levels of various hyperphosphorylated forms of Tau. Tau phosphorylation results in its dissociation from microtubules, which at normal, physiological levels helps to maintain microtubule functions, such as neurite outgrowth and axonal transport (Johnson and Stoothoff, 2004). Hyperphosphorylation, however, subsequently causes microtubule destabilization and the formation of Tau filaments. Ultimately, this results in cell death and neurodegeneration, similar to the increased apoptosis observed in the DEKsh cells. We did not observe any differences between control and DEK-deficient cells in Tau phosphorylation at sites AT180 and AT8. These sites are associated with neuronal apoptosis (Kobayashi et al., 2003) and are found to be abnormally phosphorylated in the AD brain (Schindowski et al., 2006; Neddens et al., 2018). However, we observed significantly elevated levels of phosphorylation at sites S262. Hyperphosphorylated Serine 262 is observed frequently in the AD brain and most highly inhibits Tau’s ability to bind to microtubules (Fischer et al., 2009; Haj-Yahya et al., 2020). Future work will probe the microtubule stability of neurites after DEK loss as a consequence of Tau hyperphosphorylation. Additionally, future studies will investigate the molecular mechanism(s) by which hyperphosphorylation of Tau occurs after DEK loss. Given that β-catenin levels are decreased in DEKsh cells, it is possible that GSK3β, a kinase in the Wnt/β-catenin pathway, could be deregulated with DEK loss. GSK3β is known to phosphorylate Tau at multiple sites that are linked with AD (Hanger et al., 2009), and this is associated with inhibited binding of Tau to microtubules (Wagner et al., 1996).

Given the neurite insufficiency and increased apoptosis noted in DEKsh cells, it is evident that differentiated neuronal cells experience significant amounts of cellular stress in the absence of DEK protein. This is further supported by the increased levels of phosphorylated p53, a marker of cell stress, in DEK-deficient differentiated SH-SY5Y cells. However, p53 phosphorylation is also a marker of DNA damage. DEK loss previously has been correlated with impaired non-homologous end-joining and homologous recombination mechanisms of DNA double-strand break repair (Kavanaugh et al., 2011; Smith et al., 2017). The role of DNA damage in AD pathogenesis is of growing interest, and several groups have provided evidence that impaired DNA damage repair is associated with the disease (Coppedè and Migliore, 2009; Shanbhag et al., 2019). Also, activated p53 is found to be upregulated in AD brains and indirectly induces Tau phosphorylation *in vitro* (Hooper et al., 2007; Proctor and Gray, 2010). Cellular stress promotes the translocation of Tau to the nucleus (Sultan et al., 2011; Violet et al., 2014), where it can preserve DNA integrity in neurons (Sultan et al., 2011; Violet et al., 2014; Bukar Maina et al., 2016). Given the role of DEK in DNA damage repair (Kavanaugh et al., 2011; Smith et al., 2017), additional work is needed to determine if nuclear Tau and DEK cooperate to maintain genome stability in neurons, or if nuclear localization of Tau, and its hyperphosphorylation, are induced to mitigate the genomic stress caused by insufficient DNA damage repair in DEK deficient cells. Studying the relationship between DEK loss and nuclear Tau with the onset of AD pathology could provide more insight into the role of DNA damage in AD and neurodegeneration. Thus, additional work is needed to determine if the apoptosis of neuronal cells induced by DEK loss is due to stress caused by impaired microtubule dynamics or DNA damage, or both.

Previously, we reported that DEK is expressed in hippocampal neurons and that DEK protein levels are lower in the brains of elderly women with dementia, but not age-matched men. Here, we have identified DEK as a novel player in dementia and AD by showing that DEK loss could lead to hyperphosphorylated Tau accumulation and apoptosis. Although the pathological impact of Tau tangles vs. β-Amyloid plaques is debated, there is strong evidence to suggest that Tau hyperphosphorylation is a major factor in AD development (Giacobini and Gold, 2013; Kametani and Hasegawa, 2018; van der Kant et al., 2020). This research could assist in a deeper understanding of the molecular mechanisms underlying AD, as well as potential therapeutic targets.

## Data Availability Statement

The original contributions presented in the study are included in the article/Supplementary Materials, further inquiries can be directed to the corresponding author.

## Author Contributions

AG, LP, and LPV carried out data collection and data analysis. AG wrote the manuscript with support and input from MS and LPV. All authors contributed to the article and approved the submitted version.

## Conflict of Interest

The authors declare that the research was conducted in the absence of any commercial or financial relationships that could be construed as a potential conflict of interest.

## Supplementary Material

SUPPLEMENTARY FIGURE 1 Sites AT180 and AT8 of Tau are not abnormally phosphorylated in DEKsh cells. **(A–C)** DEKsh leads to increased unphosphorylated Tau (Tau-1 antibody) protein expression, quantified *via* Western blot (**A,B**; ***p* < 0.01) and immunofluorescence **(C)**. **(D)** Representative immunofluorescence images demonstrate increased unphosphorylated Tau (Tau-1) in DEKsh cells. Representative images of phosphorylated Tau at sites AT180 and AT8. **(E)** Fluorescent intensity analysis reveals no significant difference in Tau phosphorylation at AT180 or AT8 in DEKsh cells.
