# Supplementary figures and images for: Loss of DEK Expression Induces Alzheimer’s Disease Phenotypes in Differentiated SH-SY5Y Cells
Source: Front Mol Neurosci. 2020 Nov 16;13:594319. doi: 10.3389/fnmol.2020.594319 (PMC7701170; doi:10.3389/fnmol.2020.594319)

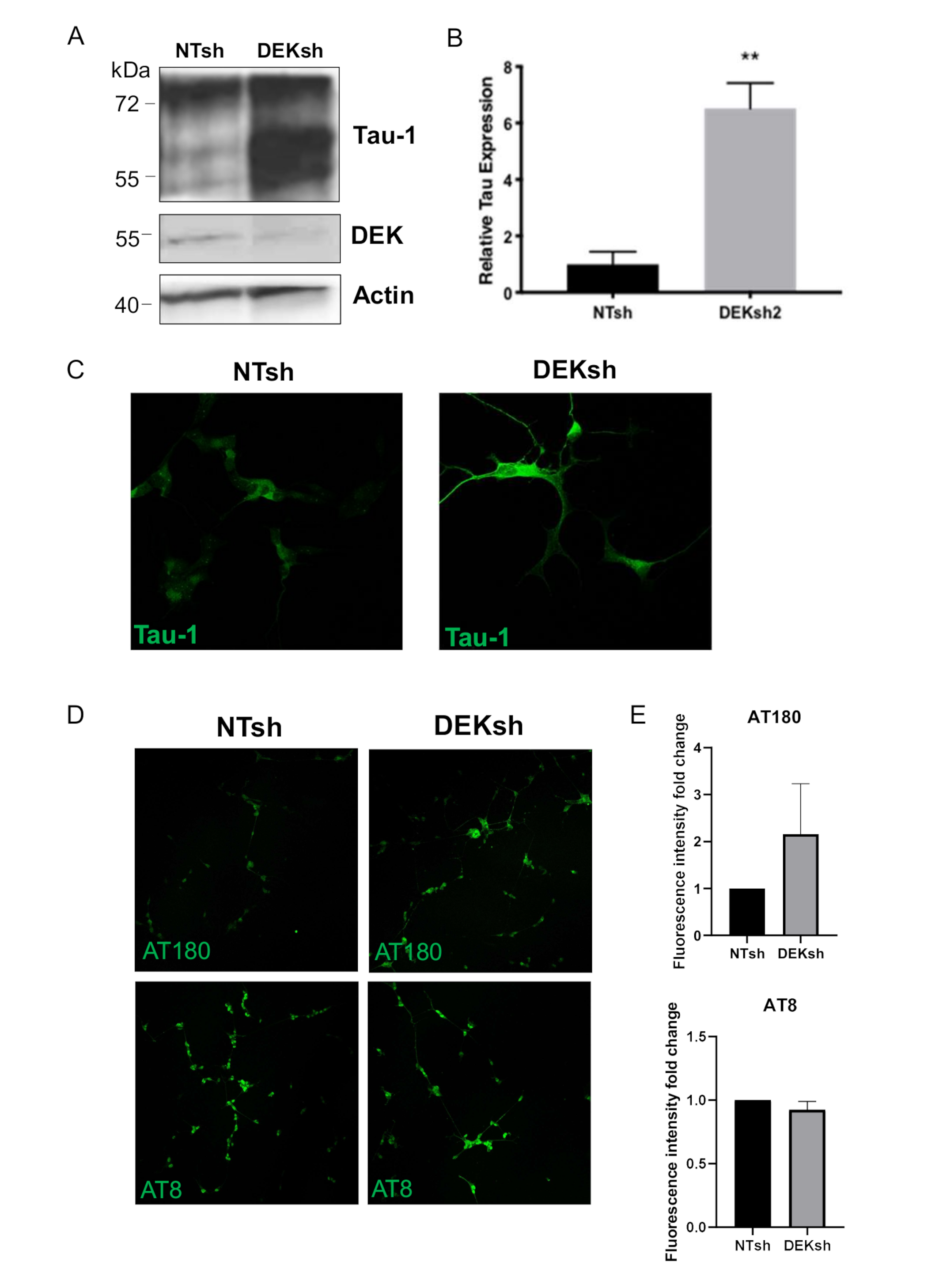

Supplement: SUPPLEMENTARY FIGURE 1 — Sites AT180 and AT8 of Tau are not abnormally phosphorylated in DEKsh cells. (A–C) DEKsh leads to increased unphosphorylated Tau (Tau-1 antibody) protein expression, quantified via Western blot (A,B; **p < 0.01) and immunofluorescence (C). (D) Representative immunofluorescence images demonstrate increased unphosphorylated Tau (Tau-1) in DEKsh cells. Representative images of phosphorylated Tau at sites AT180 and AT8. (E) Fluorescent intensity analysis reveals no significant difference in Tau phosphorylation at AT180 or AT8 in DEKsh cells. [file Image_1.TIF]
